# Harnessing cross-species alignment to discover SNPs and generate a draft genome sequence of a bighorn sheep (*Ovis canadensis*)

**DOI:** 10.1186/s12864-015-1618-x

**Published:** 2015-05-20

**Authors:** Joshua M Miller, Stephen S Moore, Paul Stothard, Xiaoping Liao, David W Coltman

**Affiliations:** Department of Biological Science, University of Alberta, Edmonton, Alberta Canada; Centre for Animal Science, Queensland Alliance for Agriculture & Food Innovation, University of Queensland, St Lucia, QLD Australia; Department of Agricultural, Food and Nutritional Science, University of Alberta, Edmonton, Alberta Canada; Tianjin Institute of Industrial Biotechnology, Chinese Academy of Sciences, Tianjin, China

**Keywords:** Cross-species alignment, Comparative genomics, Domestication

## Abstract

**Background:**

Whole genome sequences (WGS) have proliferated as sequencing technology continues to improve and costs decline. While many WGS of model or domestic organisms have been produced, a growing number of non-model species are also being sequenced. In the absence of a reference, construction of a genome sequence necessitates *de novo* assembly which may be beyond the ability of many labs due to the large volumes of raw sequence data and extensive bioinformatics required. In contrast, the presence of a reference WGS allows for alignment which is more tractable than assembly. Recent work has highlighted that the reference need not come from the same species, potentially enabling a wide array of species WGS to be constructed using cross-species alignment. Here we report on the creation a draft WGS from a single bighorn sheep (*Ovis canadensis*) using alignment to the closely related domestic sheep (*Ovis aries*).

**Results:**

Two sequencing libraries on SOLiD platforms yielded over 865 million reads, and combined alignment to the domestic sheep reference resulted in a nearly complete sequence (95% coverage of the reference) at an average of 12x read depth (104 SD). From this we discovered over 15 million variants and annotated them relative to the domestic sheep reference. We then conducted an enrichment analysis of those SNPs showing fixed differences between the reference and sequenced individual and found significant differences in a number of gene ontology (GO) terms, including those associated with reproduction, muscle properties, and bone deposition.

**Conclusion:**

Our results demonstrate that cross-species alignment enables the creation of novel WGS for non-model organisms. The bighorn sheep WGS will provide a resource for future resequencing studies or comparative genomics.

**Electronic supplementary material:**

The online version of this article (doi:10.1186/s12864-015-1618-x) contains supplementary material, which is available to authorized users.

## Background

Widespread use of high-throughput sequencers has allowed an ever increasing number of species to have a whole genome sequence (WGS) prepared. While many of these have been model or domestic organisms, a wide array of taxa continue to be sequenced (as reviewed in [1]). WGS opens the door for a multitude of subsequent analyses including: 1) creation of phylogenies and assessment of broader evolutionary patterns and innovations [2, 3]. 2) Annotation of genes [4] and identification of rearrangements or gene expansions [5, 6]. 3) Discovery of large sets of markers [7, 8]. 4) Resequencing studies, including those that are genome-wide yet not full coverage (e.g. transcriptomics or reduced representation sequencing) but benefit from the presence of a reference genome [9]. Resequencing at any scale also allows for ‘population genomics’ including investigations of local adaptation or population differentiation [10, 11], demographic history [12, 13], and the genetic basis of phenotypic traits [14].

In the absence of a reference, construction of a WGS necessitates *de novo* methodologies [15]. These methods require large volumes of raw sequence data which are arranged into contigs and then joined to scaffolds by either computational methods [16], anchoring with outside information (e.g. a linkage map, BACs, or FISH), or continued sequencing [17]. Such an endeavor is still relatively expensive and challenging in terms of the bioinformatics involved, making it beyond the capability of many research programs. However, the presence of a reference sequence enables reads to be aligned to the reference which is much faster and allows for lower sequence depths than *de novo* assembly [17, 18]. Recent work has highlighted that the reference need not come from the same species the reads are from [19–22] opening these methods to a wide array of ‘genome-enabled’ taxa [23].

There are a number of reasons why we are motivated to produce a bighorn sheep (*Ovis canadensis*) WGS. First, this species has a complex demographic history in North America that has been profoundly influenced by anthropogenic activity, having experienced intense hunting, local extirpations and disease-related die-offs, as well as translocations and reintroductions throughout its range [24–29]. These events are expected to have significant genetic/genomic consequences [26, 28, 30] that merit further study. Second, there are several long-term study populations in which individual based questions such as the genetic basis of complex traits [31, 32] and linkages between individual genetic variation and differences in fitness [33, 34] can be addressed using genomic data. Finally, bighorn sheep are an excellent candidate for cross-species approaches since genomic resources for domestic sheep (*Ovis aries*, [35, 36]) can be easily applied to bighorn sheep as they are a close relative (~3 million years divergent; [37]) and are expected to share a high degree of genomic synteny [38]. Genomic resources have been recently developed for bighorn sheep [38–42], but future resequencing efforts would be aided by species specific genomic sequence data.

Here we use cross-species alignment to create a draft genome from a single ram sequenced using ABI SOLiD technology. The pros and cons of different high-throughput sequencers have been discussed at length elsewhere [43–46]. Choice of a specific platform balances read length, the amount of sequence data output, error profiles, and cost. SOLiD technology is well-suited for resequencing studies as it returns high volumes of data and the sequence-by-ligation strategy is able to distinguish sequencing errors from true nucleotide variants during alignment [47, 48]. Based on our alignment we called variants, annotated SNPs relative to domestic sheep, and conducted enrichment analysis of those SNPs showing fixed differences.

## Results

### SOLiD sequencing and alignment

Whole-genome sequencing of a single bighorn sheep ram was performed using two libraries and ABI SOLiD platforms. Prior to trimming the 50 × 50 bp mate-paired library contained 311,847,628 reads, while the 75 bp fragment library contained 555,575,794 reads. Filtering and alignment were then conducted on both libraries in CLC Genomics Workbench (version 5.0). Post-trimming, read count was 218,239,459 (70% retained) and 506,697,724 (91% retained) for the mate-paired and fragment libraries respectively.

The resulting reads from each library were then independently aligned to domestic sheep chromosomes version 3.1 [36]. When aligned on its own the mate-paired library had 174,894,731 reads map to the reference, of which 115,727,618 were in pairs with an average distance of 1108 nucleotides between pairs, while the fragment library had 377,008,050 reads map to the reference. Once merged, the two libraries covered 95% of the reference genome with an average read depth of 12 (104 SD).

### Variant calling

In total, 15,622,884 variants (14,583,355 SNPs and 1,039,529 indels) passed our filtering thresholds (quality ≥30, read depth between 6 and 200) and were called compared to the domestic sheep reference using SAMtools version 0.1.17 [49]. Of the putatively bi-allelic SNPs relative to the domestic sheep reference, 9,831,700 were transitions and 4,320,985 were transversions (ti/tv = 2.275; which is similar to the 2.1 ratio observed for genomic data in many mammalian studies [50]). Insertions were slightly more common than deletions (Additional File 1). To assess SNP calling accuracy, genotypes from the aligned genome were compared to those generated for the same individual on the Ovine Infinium®HD SNP BeadChip [35]. Of the 606,006 loci present on the array 422,975 loci were successfully genotyped in our bighorn sheep sample. Note that a decrease in amplification success is expected from cross-species application of SNP chips [51, 52]. 407,465 (~96%) of these chip loci were present in the list of variants identified by sequencing, and over 93% of the loci showed agreement (Table 1). To assess the effects of filtering on these results an additional set of filtering criteria was applied to the sequence-derived SNPs. Increasing our stringency thresholds for SNPs in the WGS decreased the number chip loci that were present in the list of SNPs identified by sequencing (n = 329,690; ~78%), but increased concordance to ~95%. In both cases the major source of discordance was loci called heterozygous in the WGS but homozygous from the chip data (Table 1).

**Table 1:** Number of loci showing concordance or discordance between the genome and the Ovine Infinium®HD SNP BeadChip

	Original filter	Stringent filter
Same genotype	377129	314734
Heterozygous on chip, Homozygous in sequence	456	130
Homozygous on chip, Heterozygous in sequence	22837	9565
Alternate homozygotes	7169	5261

### Annotation and enrichment analysis

**Fig. 1:**
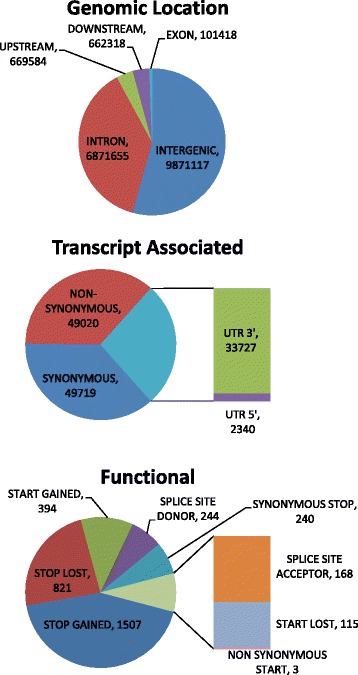
Distribution of SNP annotations and effect predictions

SnpEff [53] assigned 18,176,092 functional classes to our SNPs based on annotation of the domestic sheep genome. Note that the number of classes assigned is larger than the number of loci due to the fact that the categories are not mutually exclusive. The vast majority of the SNPs were predicted to be intronic or intergenic and 102,231 were assigned to coding regions or have predicted functional effects (Fig. 1, Additional File 2). Of those 102,231 loci, 52,381 SNPs were found to have fixed differences between our bighorn sheep and the domestic sheep reference, from which 25,472 were identified as non-synonymous and 27,198 were identified as synonymous. Note that sum of the number of synonymous and non-synonymous SNPs is larger than the total number of fixed differences because a locus may be classified as both synonymous and non-synonymous if a gene has more than one annotated transcript. Gene Ontology (GO) terms were available for 26,629 of the SNPs with fixed differences (9,752 non-synonymous and 16,877 synonymous) representing 6,963 genes (3,948 non-synonymous and 5,932 synonymous). We looked for functional enrichment between non-synonymous and synonymous SNPs using BLAST2GO [54]. When reduced to the most specific GO terms, we found 11 biological process GO terms to be over represented and 29 to be underrepresented in the non-synonymous set compared to the synonymous set (Additional File 3). Note that gene length was positively correlated to the number of annotated loci for both the non-synonymous and synonymous sets (r = 0.43 and 0.61 respectively). But given that this association was constant between both non-synonymous and synonymous gene sets we do not think it biases our results. However, one gene, titin, was ~3 times larger than all other genes considered so we repeated the GO enrichment analysis dropping titin, which reduced the level of correlation (r = 0.37 and 0.51 respectively). When titin is removed from the datasets the number of overrepresented and underrepresented terms decrease to 9 and 15 respectively; all of which were common to the set including titin, except for one underrepresented term (cellular protein metabolic process; GO 0044267) that was unique to the second analysis (Additional File 3).

## Discussion

Here we present a draft bighorn sheep WGS created by cross-species alignment to a domestic sheep reference sequence. Other studies have attempted *de novo* assembly with SOLiD sequencing data [55–57], but this was not an option in our case due to the high read depth required by such methods for a mammalian sized genome. Our work more closely resembles that of Canavez *et al.* and [22] Wang *et al.* [19]. Canavez *et al*. created a draft genome for an indicine bull (*Bos indicus*) through alignment of SOLiD reads to a taurine cow (*Bos taurus*) reference genome (divergence ~250 kya) [22]. While Wang *et al*. used SOLiD sequencing in a reference guided assembly of a black grouse (*Tetrao tetrix*) draft genome. However, Wang *et al.* [19] used a combination of *de novo* and alignment methods as the large divergence time between black grouse and domestic chicken (*Gallus gallus*) used as a reference (~30-40 mya) may hinder sequences from aligning properly. In contrast, bighorn and domestic are much less divergent which allows for successful direct alignment of reads: over 76% of our quality filtered reads mapped to the reference genome. Once merged, our two sequencing libraries provided 95% coverage of the reference and average 12x (104 SD) sequence depth.

Our alignment produced a large database of SNP markers for future studies. Approximately 6% of genotypes from a high-density SNP chip were discordant with those from the genome alignment, and increasing the quality thresholds for loci discovered in the genome only marginally decreased mismatches to ~4%. In both cases the major source of discordance was loci called heterozygous in the genome alignment but homozygous from the SNP chip. This source of discordance could be caused by incorrect joining of paralogous regions due to our procedure of randomly mapping ambiguous alignments. However, given the overall high concordance between the genome aligned SNPs and those on the SNP chip we are confident that the majority of our genotypes represent real SNPs. These markers add to the set of SNPs already available for this species [39, 42].

Genome scans of domestic sheep breeds have shown a number of regions that have been differentiated due to domestication [36, 58]. Therefore, we expect alleles associated with production traits to have been swept to or near fixation relative to a wild ancestor as well. Our GO term analysis of fixed SNP differences compared to the domestic sheep reference highlighted 40 biological process GO terms with significantly different representation in SNPs tagged as non-synonymous versus synonymous. Two of the gene ontologies that were associated with amino-acid changes relative to the domestic sheep reference involved reproduction: spermatogenesis (GO:0007283), and negative regulation of mammary gland epithelial cell proliferation (GO:0033600). This mirrors recent work has highlighted the genetic effects domestication had on reproductive traits of several sheep breeds [58, 59]. Another term that was over-represented in the non-synonymous gene set was ossification involved in bone maturation (GO:0043931). This term is noteworthy given the relationship of bones to horns which are bony projections covered by a keratinous sheath [60]. Horns are a major determinant of reproductive success in bighorn sheep, where larger males with bigger horns win antagonistic encounters and gain access to females [61, 62]; however, in many breeds of domestic sheep horns have been selected against leading to gene-level consequences [58]. All but two of the overrepresented biological process terms (skeletal muscle adaptation (GO:0043501) and maintenance of fidelity involved in DNA-dependent DNA replication (GO:0045005)) remained significant when titin (the largest gene in the dataset) was removed from the analysis.

For genes less likely to have amino acid changes, 14 of the 29 GO terms were related to muscles or muscle fibers, particularly cardiac muscles, e.g.: cardiac muscle hypertrophy (GO:0003300), cardiac myofibril assembly (GO:0055003), cardiac muscle fiber development (GO:0048739), adult heart development (GO:0007512), regulation of relaxation of cardiac muscle (GO:1901897), sarcomerogenesis (GO:0048769). It is interesting to note these differences associated with muscle properties, given that the domestic sheep reference genome was built from a meat-producing breed, the Texel [36, 63]. As mentioned above, body size is an important life history characteristic for male bighorn sheep as it relates to access to females, while larger females have been found to have longer lifespans [64]. Selective breeding for meat production in domestic sheep could favor conservation of the genes or developmental pathways that produce large muscles in bighorn sheep. However, analysis with REVIGO [65] indicated that there was overlap in these GO terms with 10 terms falling into two more representative terms: cardiac muscle hypertrophy (GO:0003300; containing two other terms) and cardiac muscle tissue morphogenesis (GO:0055008; containing eight other terms). In addition, nine of these terms become non-significant when titin (which has known associations with muscle properties, including body size, in cattle Bos taurus; [66, 67] and pigs Sus scrofa; [68]) is removed from the datasets.

Two factors are important to keep in mind when interpreting the results of our GO analysis. The first is that though it is tempting attribute the majority of differences we observed here to domestication and selective breeding, there are likely to be additional factors at play. In particular, natural selection as bighorn sheep and the progenitor to domestic sheep diverged, as well as genetic drift. Second, we are only comparing SNP sites from one individual’s genome to a reference sequence. This likely results in missing polymorphisms within either species, leading to incorrect annotation of fixed differences. However, we present the results only as a preliminary analysis to highlight candidate ontologies that may contribute to differentiation between the species. Such results will need to be confirmed by additional sequencing, alternate analyses (e.g. genome scans), and perhaps functional characterization [69].

While our draft genome represents a step forward in the genomic resources available for bighorn sheep this single genome is representative of a specific demographic history, an example of the ‘n = 1 constraint’ [70]. Future population genomic studies using additional individuals from Ram Mountain or other populations can confirm the variants we describe here, discover additional variants, and more fully examine the demographic history of bighorn sheep [71]. Expanded sequencing efforts would also allow for comparative genomics to further identify ancestral states and regions of selection relative to domestic sheep. In addition, our bighorn sheep genome can aid reference guided genome assembly [20–22] of other *Ovis* species using a genome that has not been subject to strong selective breeding.

## Conclusion

In this study, we created a WGS for bighorn sheep using the closely related domestic sheep as a reference for alignment. This procedure was highly successful, covering 95% of the reference with an average read depth of 12 (104 SD). From this sequence we were able to call 15,622,848 variants and found 40 GO terms with significantly different representation in fixed SNPs tagged as non-synonymous versus synonymous. We hypothesize that these differences may largely be a result of selection during domestication. Our results demonstrate that cross-species alignment enables the creation of novel WGS for non-model organisms. The bighorn sheep WGS will provide a resource for future resequencing studies or comparative genomics both for other populations of bighorn sheep or species within the Ovis genus.

## Methods

### Sample collection & sequencing

Total genomic DNA was extracted from tissue of a single bighorn sheep from Ram Mountain (Alberta, Canada), using standard phenol–chloroform extraction protocols [72]. From this, two libraries were constructed and sequenced. The first was a mate-paired library the details of which are provided in [40]. Briefly, preparation used the reagents and protocols provided by Applied Biosystems with and an expected insert size of ~1.5 kb. Emulsion PCR was performed using the SOLiD EZ bead system (Life Technologies Corporation). Both forward and reverse tags were sequenced to 50 bases using an Applied Biosystems SOLiD 4 sequencer (Life Technologies Corporation). The second library was a fragment library sequenced to 75 bases using a SOLiD 5500xl sequencer (Life Technologies Corporation). The resulting xsq files were converted to csfasta and qual scores format using XSQ Tool (Life Technologies Corporation).

### Alignment & variant calling

Sequence quality assessment and alignment were conducted with CLC Genomics Workbench (version 5.0; CLC bio, Cambridge, MA, USA). For each library, sequences were quality trimmed allowing for 1 ambiguous nucleotide, a quality score limit of 0.05, and minimum read length of 15 nucleotides. The resulting reads from each library were then independently aligned to domestic sheep chromosomes (version 3.1; [36]). Alignment parameters were set with no reference masking, mismatch cost of 2, insertion/deletion cost of 3, length fraction of 0.5, and similarity fraction of 0.8. Meaning at least 50% of a read must have at least 90% identity to the reference to be aligned. Non-specific matches were mapped randomly. Once mapped, PCR duplicates were removed from the alignment. We then merged the mate-paired and fragment mappings and removed PCR duplicates from the merged file. The merged alignment was then exported both as consensus fasta sequences as well as a BAM file for use in subsequent analyses. When generating the consensus fasta sequences we allowed for ambiguities (e.g. IUPAC codes W, R, etc.) and inserted N’s proportional to the length of the domestic sheep reference for regions of zero coverage. We elected to leave differences between our bighorn sheep sequence and domestic sheep reference as ambiguities in case additional sequencing reveals those sites to represent unobserved shared polymorphisms.

Variants were called from the consensus alignment BAM files using the mpileup command in SAMtools version 0.1.17 [49] and filtered in bcftools. Specifically, variants were required to have a minimum quality of 30 and a read depth between 6 and 200. VCFfilter version 0.1.11 [73] was then used to assess indel length distribution and calculate transition transversion (ti/tv) ratio using 100 basepair windows. As a quality check, genotypes from the aligned genome were compared to those generated for the same individual on the Ovine Infinium®HD SNP BeadChip, a newly developed SNP array for domestic sheep that contains 606,006 loci [35]. For this analysis raw intensity data were converted into genotype calls using GenomeStudio (Illumina) and SNP cluster information based on domestic sheep reference samples provided by the International Sheep Genomics Consortium. All genotype calls with GenCall scores less than 0.6, or GenTrain scores lower than 0.8, were removed from the data set. When assessing concordance between genotypes from the SNP array and the draft WGS we first positioned SNPs from the array in the reference assembly by comparing 50 nucleotides on either side of the locus position using BLAST with an E value of 1e^−9^. Loci with more than one match were excluded from analysis. In total this procedure excluded 45,979 loci. To assess the effects of filtering on the recovery of chip SNPs by sequencing and on concordance between the chip and the sequence genotypes an additional set of filtering criteria was applied to the sequence-derived SNPs. In this case we increased stringency, requiring read depths greater than 5 but less than the mean plus 3 SD, at least one forward or reverse alternative allele read (where applicable), no other variants within 5 bp, and genotype quality greater than 10.

### Annotation and enrichment analysis

SnpEff version 3.1 [53] was used to predict functional classes (e.g. intergenic or intronic) and effect types (e.g. synonymous or non-synonymous) of the loci by comparing our SNPs to annotations from the domestic sheep genome (database oar3.1, downloaded Sept 2013). Note that within functional classes and effect types, categories are not mutually exclusive, for example a SNP can be classified as both intronic and in the 5’-UTR.

For enrichment analysis we first filtered SNPs to only those that were fixed between our bighorn sheep alignment and the domestic sheep reference using SNPsift [74]. We then split the resulting loci into two categories: 1) those with likely functional consequences (i.e. non-synonymous coding, start gained, start lost, stop gained, stop lost) and 2) those showing synonymous effects (i.e. synonymous coding, synonymous start). GO terms were added to the SNPs in these lists from the *Ovis aries* gene set (Oar v3.1) using BioMart [75] and Ensembl version 77 [76]. The two groups were then compared using BLAST2GO [54] which employs a Fisher’s Exact Test via the Gossip package [77]. Specifically, we used a two tailed test with false discovery correction of Benjamini and Hochberg [78] set at 0.0001. Evaluation of GO enrichment among candidate genes was restricted to terms within the biological process category.

#### Data accessibility

Raw reads have been deposited on NCBI SRA as SRR1752652 (mate-paired library) and SRR1752652 (fragment library), and genome fasta have been deposited at DDBJ/EMBL/GenBank under the accession JZLK00000000.

## Additional files

Additional file 1: **Histogram of insertion/deletion lengths in the bighorn draft genome relative to the domestic sheep reference.**

Additional file 2: **Summary of predicted effects of each SNP by chromosome as assigned by SnpEff.**

Additional file 3: **GO enrichment summary between loci predicted to be non-synonymous and those predicted to be synonymous.**
