# Supplementary figures and images for: Harnessing cross-species alignment to discover SNPs and generate a draft genome sequence of a bighorn sheep (Ovis canadensis)
Source: BMC Genomics. 2015 May 20;16(1):397. doi: 10.1186/s12864-015-1618-x (PMC4438629; doi:10.1186/s12864-015-1618-x)

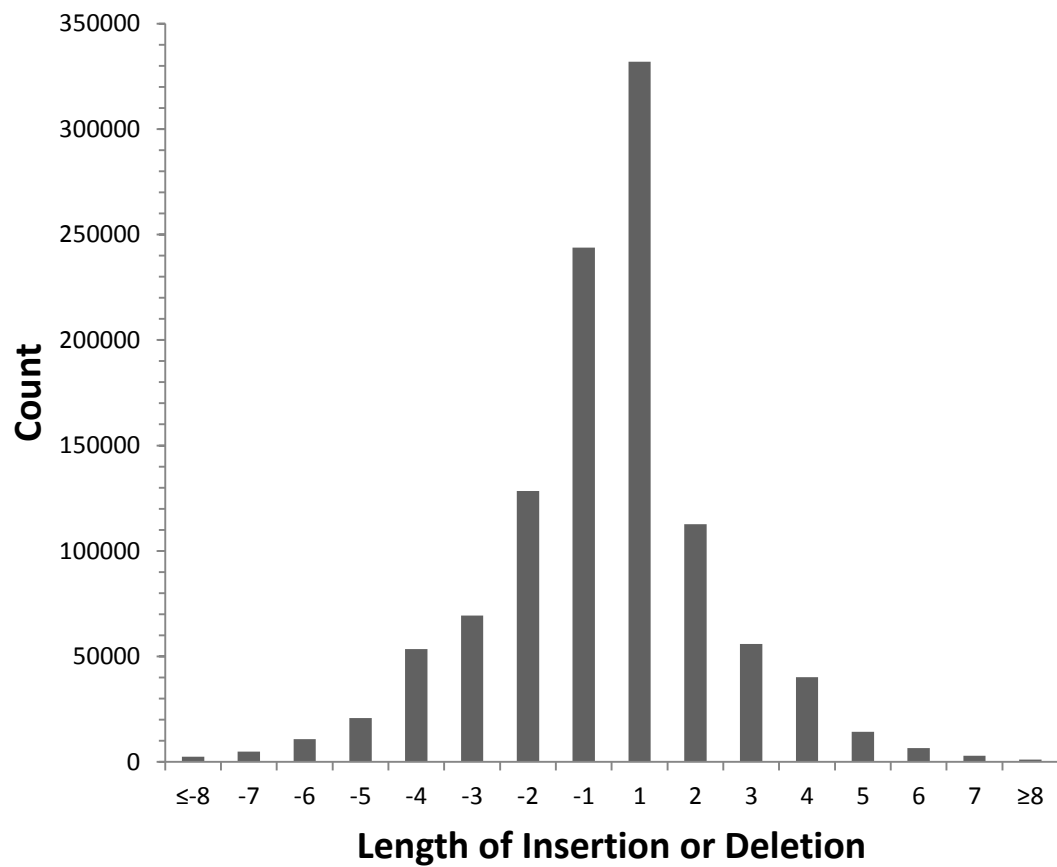

Supplement: Supplementary file 1 — Histogram of insertion/deletion lengths in the bighorn draft genome relative to the domestic sheep reference. [file 12864_2015_1618_MOESM1_ESM.pdf]
